# Functional Compartmentalization of the Human Superficial Masseter Muscle

**DOI:** 10.1371/journal.pone.0116923

**Published:** 2015-02-18

**Authors:** Rodrigo A. Guzmán-Venegas, Jorge L. Biotti Picand, Francisco J. Berral de la Rosa

**Affiliations:** 1 Laboratorio Integrativo de Biomecánica y Fisiología del Esfuerzo, Escuela de Kinesiología, Facultad de Medicina, Universidad de los Andes, Santiago, Chile; 2 Facultad de Odontología, Universidad de los Andes, Santiago, Chile; 3 Laboratorio de Biomecánica, Kinesiología y Cineantropometría, Universidad Pablo de Olavide, Sevilla, España; Max Planck Institute for Evolutionary Anthropology, GERMANY

## Abstract

Some muscles have demonstrated a differential recruitment of their motor units in relation to their location and the nature of the motor task performed; this involves functional compartmentalization. There is little evidence that demonstrates the presence of a compartmentalization of the superficial masseter muscle during biting. The aim of this study was to describe the topographic distribution of the activity of the superficial masseter (SM) muscle’s motor units using high-density surface electromyography (EMGs) at different bite force levels. Twenty healthy natural dentate participants (men: 4; women: 16; age 20±2 years; mass: 60±12 kg, height: 163±7 cm) were selected from 316 volunteers and included in this study. Using a gnathodynamometer, bites from 20 to 100% maximum voluntary bite force (MVBF) were randomly requested. Using a two-dimensional grid (four columns, six electrodes) located on the dominant SM, EMGs in the anterior, middle-anterior, middle-posterior and posterior portions were simultaneously recorded. In bite ranges from 20 to 60% MVBF, the EMG activity was higher in the anterior than in the posterior portion (p-value = 0.001).The center of mass of the EMG activity was displaced towards the posterior part when bite force increased (p-value = 0.001). The topographic distribution of EMGs was more homogeneous at high levels of MVBF (p-value = 0.001). The results of this study show that the superficial masseter is organized into three functional compartments: an anterior, a middle and a posterior compartment. However, this compartmentalization is only seen at low levels of bite force (20–60% MVBF).

## Introduction

The masseter muscle is involved in complex motor tasks such as swallowing, biting and talking. Anatomically, it consists of two portions, superficial and deep [1, 2], which function differently in mandibular movements [3]. Heterogeneity in the activation of motor units has been observed in these portions, thereby configuring functional compartmentalization [3–6]. In the deep masseter muscle, two functional compartments, an anterior and a posterior, have been clearly identified [3]. However, in the superficial masseter muscle, results have been inconclusive. One of the factors responsible for these inconclusive results could be the recording technique used. Previous studies [3, 6] have used fine-wire electrodes, which have a reduced recording area, only depicting the activity of small areas of the muscle [7]. By contrast, in high-density surface electromyography (HDEMGs) [8, 9], surface electrodes are used. These electrodes are arranged in a two-dimensional matrix that provides a broad recording area and covers much of the total area of a superficial muscle, thus making it possible to record the activity of many motor units (MUs) in different parts of the same muscle. In combination with new evidence obtained *in vivo* that supports the presence of functional compartmentalization of the human superficial masseter (SM), this research could help facilitate a better understanding of this muscle´s function in addition to the associated motor control mechanisms. This evidence could potentially be useful in the evaluation of normal and dysfunctional conditions and rehabilitation. Furthermore, availability of data regarding muscle activation of the SM in its various portions may be useful in modeling techniques when it is not feasible to collect in vivo data, such as in the case of virtual anthropology research [10–12].

The aim of this study was to describe the topographic distribution of the activity of the SM muscle motor units using non-invasive EMG at different levels of bite force (BF) to evaluate the following hypothesis: “*the* SM has a functional compartmentalization *in vivo*, as measured with *HDEMGs*, with regard to the magnitude of the BF”.

## Methods

### Participants

**Figure 1:**
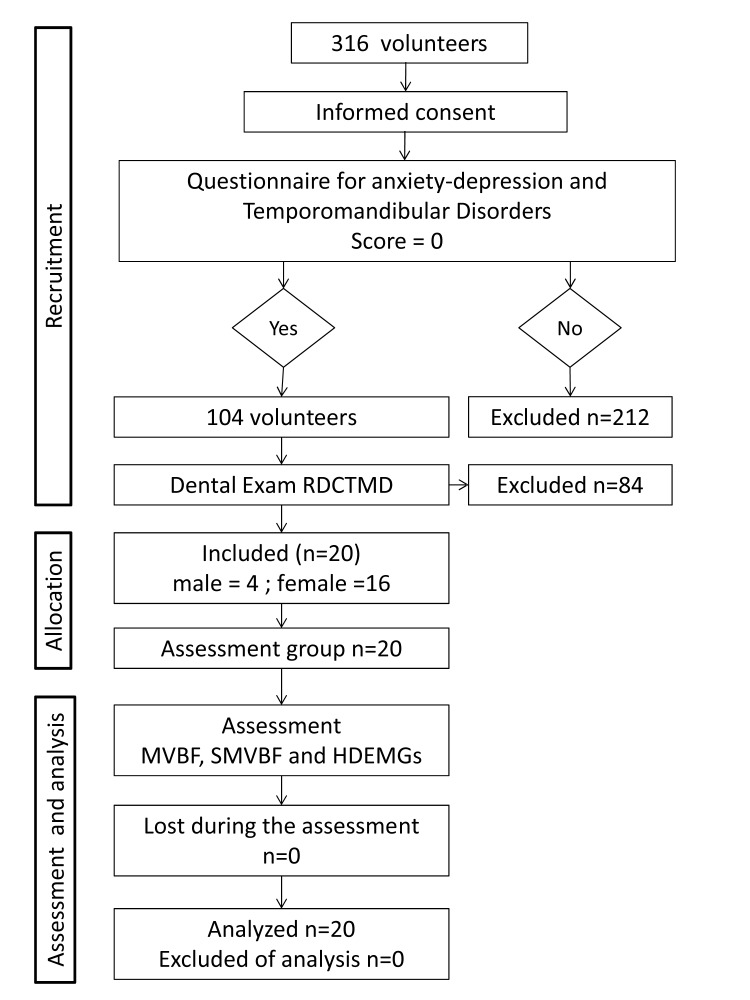
Study design. RDCTMD: Research diagnostic criteria for temporomandibular disorders. MVBF: Maximal voluntary bite force. SMVBF: Submaximal voluntary bite force. HDEMGs: High density surface electromyography.

Samples were obtained following the procedure shown in Fig. 1. All volunteers were asked to complete the Goldberg anxiety-depression questionnaires [13] and the temporomandibular disorders questionnaire [14], which were used to identify any participants who had symptoms or signs of temporomandibular disorders. A dental clinical examination based on the Research Diagnostic Criteria for Temporomandibular Disorders was applied to volunteers without symptoms and signs (Score = cero in both questionnaires) [15] to rule out the presence of temporomandibular disorders. Twenty volunteers (men: 4; women: 16; age: 20 ± 2 years old; mass: 60 ± 12 kg; height: 163 ± 7 cm; mean ± standard deviation) were selected. The Bioethics Committee of the Universidad de Los Andes approved the study. All participants provided written informed consent. The experiment was performed according to the principles and guidelines of the Declaration of Helsinki (1975).

### Experimental protocol

Each volunteer sat in a dental chair with the backrest inclined to 110° with a head support and a slight head-neck extension of 10°. Volunteers were asked to make three bites on a gnathodynamometer with maximum voluntary bite force (MVBF). Each bite lasted five seconds with one minute rest in between. The magnitude of the MVBF was defined as the maximum force value recorded in the three bites. Then, volunteers performed four submaximal voluntary bites (SMVBF) equivalent to 20, 40, 60 and 80% of the MVBF, randomly. Each repetition lasted 15 seconds, with a five minute rest period in between. Volunteers received real-time visual feedback of the dominant side BF with a bar graph displayed on a monitor in front of them to help control its magnitude. Prior to all records, participants performed a series of tests in order to become familiar with the procedure. During the maximum voluntary bite force and submaximal voluntary bites tests, both BF and EMG activity were simultaneously recorded and stored for further analysis.

### Bite Force registration

The BF was isometrically assessed at the level of first molars at an interocclusal distance (IOD) of eight millimeters using a gnathodynamometer similar to that used in a previous study [16], which contained two stainless steel handles, each one with a strain gauge type sensor (KFG-2N-120-C1–11L1M2R: One Omega Drive. Stamford. USA). Both handles were linked by an arch-shaped bridge. BF records were bilateral and simultaneously performed. The interocclusal contact surface of the gnathodynamometer was covered by leather, which has proven to facilitate a good reproducibility rate of MVBF [17]. The entire gnathodynameter was covered with a disposable polyethylene bag. To avoid cross-infections, both the leather covers and the bag were replaced for each participant’s assessment. The gnathodynamometer signals were amplified with a gain of 162 and filtered using a fourth-order Butterworth low-pass filter, 450 Hz (Nidaq2: Kinetecnics. Santiago. Chile). This device had a linear range between 0 and 1.5 kN (r² = 0.99) and was shown to have a high reproducibility rate (intraclass correlation coefficient = 0.95).

### Electromyographic record

**Figure 2:**
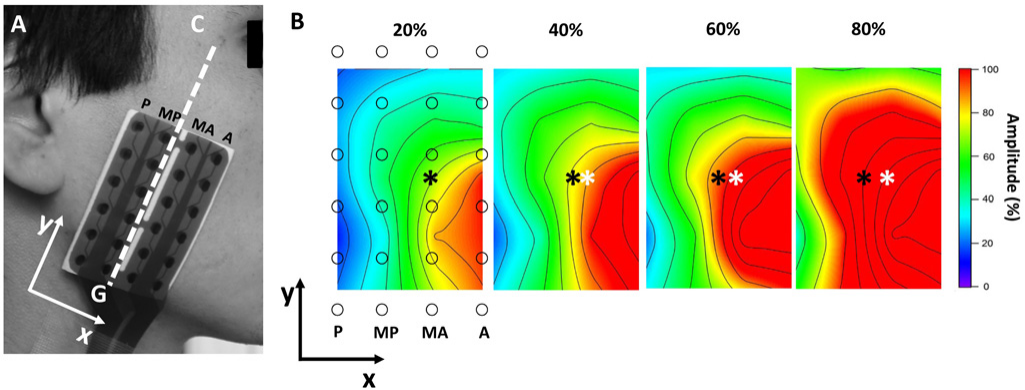
Show the matrix of electrodes used and the topographic maps of the amplitude of the EMG activity of the superficial masseter. (A) Matrix of 24 surface electrodes arranged in four columns: anterior (A), middle-anterior (MA), middle-posterior (MP) and posterior (P) columns. C: cantus, G: gonion. (B) Examples of topographic maps of the amplitude of the EMG activity of the superficial masseter recorded during bites at 20, 40, 60 and 80% of voluntary maximum bite force (VMBF). Maps were constructed in windows of 500 ms and with an interpolation factor of 8. Amplitude values of each map are expressed as a percentage of the maximum value of each one. ○: electrode positions. *: location of center of mass. *: location of center of mass at 20% of VMBF.

The dominant side, defined as the chewing side preference, was selected to obtain EMG records; on that side, the skin was cleaned with an abrasive paste (Everi: Spes Medica s.r.l. Battipagglia (SA), Italy) to improve the quality of the EMG records. The orientation of the fibers of the SM was determined by a straight line between the gonion and the cantus [18] and was corroborated by examining the direction of propagation of motor action potentials with a linear array of 16 surface electrodes (with an inter-electrode distance [IED] of 2.5 mm). Next, a flexible array of 24 surface electrodes was installed, and they were arranged in four columns of six electrodes each, with an IED of ten millimeters. The columns of the matrix were parallel to the muscle fibers. Thus, the EMG activity was recorded in four sites of the SM, which from the anterior to posterior part corresponded to the following columns: Anterior (A), Middle-Anterior (MA), Middle-Posterior (MP) and Posterior (P) (Fig. 2a). From the 24 electrodes, 20 bipolar EMG signals were amplified, distinguishing the records according to the columns. Signals were amplified with a gain of 2000 and digitized at a sampling frequency of 2048 Hz at a 12 bit resolution (EMG-USB2: OTBiolettronica Turin. Italy. 3 dB bandwidth 10–500 Hz). The matrix was fixed with a hypoallergenic adhesive, and the space between the electrodes and the skin was filled with a conductive cream (AC Cream: Spes Medica s.r.l. Battipagglia (SA). Italy).

### EMG signal Processing

All stored EMG signals were subjected offline to a second-order Butterworth digital filter with a bandwidth of 20–400 Hz (OT BioLab 1.7: OTBioelettronica. Turin. Italy). The amplitude of the signals was calculated using the root mean square (RMS). For the signals recorded during SMVBF, a window without overlap of 500 ms was used; in the signals of MVBF, a window of 50 ms was used. This window width was used to obtain a higher resolution in the search for a maximum value during MVBF. The maximum values for each one of the 20 signals recorded during the MVBF were determined. These values were considered to be the maximum EMG amplitudes produced voluntarily. Subsequently, the 20 signals recorded during the bites at SMVBF were normalized to their respective maximum values and expressed as percentages. For analysis of the signals recorded during the SMVBF, only the central five seconds from the recorded 15 seconds were considered. This was done to obtain data in a stable state; thus, fluctuations associated with reaching the force level during the first five seconds and the possible effects of muscle fatigue during the final five seconds were discarded. For the 20 signals, in each one of the BF levels, ten normalized EMG amplitude values (corresponding to the five seconds of analysis) were obtained. These ten values were averaged for each signal, and the averages of the five signals corresponding to each column were then averaged. This was done for each BF level, providing a normalized amplitude value representing the level of activation of motor units pertinent to the territory of each column (A, MA, MP and P). To describe changes in the topographical distribution of the SM EMG activity at different bite levels, the position of a center of mass (COM) was calculated from the normalized amplitude values, and the anterior-posterior (COMx) and cephalocaudal (COMy) positions were considered. A modified entropy index [19] was used to describe the uniformity degree in the distribution of the EMG activity. The entropy index measures the uniformity of a data set in arbitrary units. This index is maximized when the data of the set have the same values, describing a consistency between them. When the index is at the minimum, there is excessive variation in the data. Therefore, this index was used to characterize the level of homogeneity or heterogeneity between the activation levels of the different regions of the SM. Farina et al. [19] have described the entropy rate ranges between five and six arbitrary units.

Topographic maps were constructed to describe the distribution of the EMG activity in the SM (Fig. 2b). The maps were constructed with the RMS amplitude values standard for each channel and with the position coordinates of the channels within the grid of electrodes. Both data sets were multidirectionally interpolated with an eight factor. This procedure was performed using a signals analysis software application (IgorPro 6.0: WaveMetrics Inc. Portland, USA).

### Statistical analysis

A descriptive statistical analysis (mean and standard deviation) of the normalized amplitude variables of each column (A, MA, MP and P) of the COMx and COMy positions, the entropy indices, and the MVBF was performed. Moreover, the type of distribution of these variables was studied using the Shapiro-Wilk test. To determine the possible differences in the level of activation of the various portions of the SM, a comparison of the amplitude values between the four columns within each SMVBF level was performed. Entropy values and COMx and COMy positions between the different SMVBF levels were also contrasted. All comparisons were performed by using a one-way analysis of variance and a pairwise Bonferroni post hoc test. All analyses were performed as two-tailed tests with a significance level of 95% (STATA/SE 12.1. StataCorp. College Station. USA).

## Results

**Figure 3:**
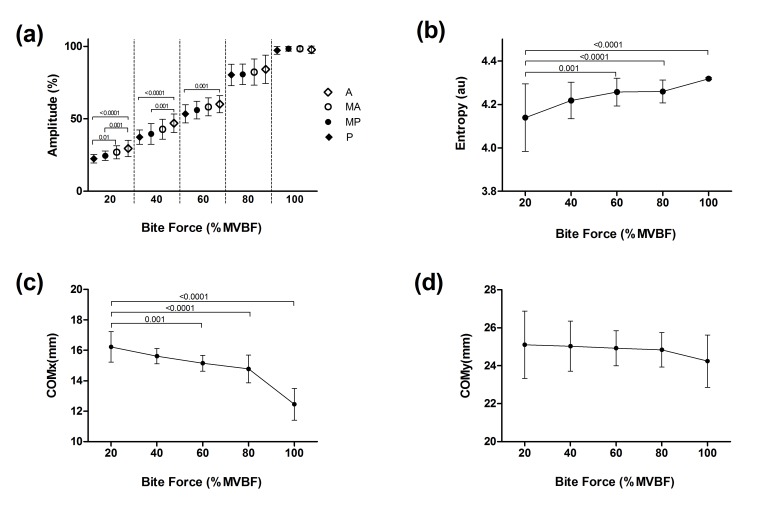
Masseter muscle electromyographic parameters at different levels of masticatory force. a) Amplitude in columns: Anterior (A), Middle-Anterior (MA), Middle-Posterior (MP) and Posterior (P). b) Values of entropy. c) Position of the center of mass in X. d) position of the center of mass in Y. The level of masticatory force is expressed as a percentage of MVBF. The differences are indicated by bars with their respective p-values.

All analyzed variables showed a normal distribution (p <0.0001), and they are expressed as their means and standard deviations (SD), as shown in Table 1. Fig. 3a shows the normalized EMG amplitude values for the anterior (A), middle-anterior (MA), middle-posterior (MP) and posterior (P) columns at different percentages of MVBF. At the level of 20%, the amplitude for A was higher in relation to the posterior columns, P (p <0.0001) and MP (p = 0.001). In this same parameter, MA showed a higher value than P (p = 0.01). At 40% MVBF, A also showed a higher amplitude than P (p <0.0001) and MP (p = 0.001). For the 60% MVBF, the amplitude of A was higher than the amplitude of P (p = 0.001). The activation difference between A and P is shown in Fig. 3a. COMx was displaced to the posterior part of the SM as the percentage of the MVBF increased. COMx positions at 60% (p = 0.001), 80% (p <0.0001) and 100% MVBF (p <0.0001) were more posterior than those recorded at 20% (Fig. 3c). For COMy, no significant changes were observed. The average MVBF was 292 Newton (SD = 77 N). Entropy indices recorded at 60% (p = 0.001), 80% (p <0.0001) and 100% MVBF (p <0.0001) increased in comparison to that observed at 20% (Fig. 3b).

**Table 1:** Means and standard deviation of normalized electromyographic amplitude for four recording sites of the superficial masseter muscle during bites at different percentages of maximal voluntary bite force by healthy volunteers (n = 20).

Columns	20%	40%	60%	80%	100%
Anterior	29.5(5.6)^A^ ^,^ ^B^	46.9(6.4)^A^ ^,^ ^B^	60.1(6.0)^A^	84.1(9.8)	97.7(2.5)
Middle-Anterior	26.9(4.4)^C^	42.8(6.8)	58.2(6.2)	82.2(9.0)	98.3(1.6)
Middle-Posterior	24.5(3.2)^B^	39.5(7.1)^B^	56.0(6.1)	80.6(7.1)	98.4(1.6)
Posterior	22.4(2.9)^A^ ^,^ ^C^	37.3(4.9)^A^	53.4(6.3)^A^	80.3(7.4)	97.3(2.7)

%: percent of maximal voluntary bite force.

^A^Statistically significant difference between the Anterior and Posterior columns (p-value <0.05).

^B^Statistically significant difference between the Anterior and Middle-posterior columns (p-value = 0.001).

^C^Statistically significant difference between the Middle-anterior and Posterior columns (p-value = 0.01).

## Discussion

This study showed a difference in the motor unit’s recruitment of the anterior and posterior portions of the SM during bite at low levels of submaximal force. This result supports the presence of functional compartmentalization of the SM.

Our results indicate that during a bite at a low level of MVBF (20–60%), the MUs located in the anterior parts of the SM are recruited more than those of the posterior parts. From the biomechanical point of view, during a static bite with a vertical dimension close to the intercuspal position, the axis of jaw rotation is located in the condyles of the temporomandibular joints. Therefore, the MUs of the anterior portion have a stronger lever arm than the posterior MUs [6, 20]. This factor provides a mechanical advantage to the anterior MUs, and possibly, for that reason, they have a lower activation threshold. In this study, the IOD was constant; therefore, we cannot guarantee that the behavior of the anterior MUs in relation to posterior MUs is maintained at different IODs, as recently observed using bipolar electrodes [21]. Furthermore, one bite at a submaximal level requires a finer control of force development. The results of this study show that during submaximal levels of BF, the EMG activity was higher in the anterior part than in the posterior part. This EMG activity is proportional to the number of active MUs. Thus, at low levels of BF, anterior MUs are recruited more than posterior ones. The higher activation of these MUs may be interpreted as a greater participation of these MUs in performing fine motor tasks [22]. Possibly, at low levels of BF, the function of the posterior portion of the SM is related to mandibular stabilization; however, this should be investigated in further studies. One finding from this study that supports this idea is that when the bite is taken to maximum levels of 80–100% MVBF (i.e., a grosser control task is performed), no differences in the recruitment of MUs between regions of the SM were observed.

Differences in the levels of activity between the superficial and deep region of the human masseter muscle during different tasks have been observed. The contribution of MUs in producing bite force is not uniform in the masseter muscle; different activation patterns between the jaw muscles and also between the superficial and deep masseter muscles are observed [23]. Furthermore, in data recently obtained by the authors of this study (unpublished) in relation to the SM, variation in recruitment of the anterior and posterior MUs during chewing of different types of food was observed, demonstrating that the compartmentalization observed in SM during submaximal static contractions persists in isotonic contractions.

The composition of the fibers of the jaw-elevator muscles has undergone evolutionary changes in vertebrates. These changes have an adaptive origin depending on the diet of each species [24]. In higher mammals, when comparing modern humans, great apes and monkeys, there is a large difference in the size of the masticatory muscles. At the histological level, it has been observed that the diameter of the type I muscle fibers between humans and monkeys (*Macaca fascicularis*) do not differ in diameter [25]. Nevertheless, type II fibers from humans would only have 1/8 of the diameter observed in these monkeys [25]. Thus, the mutation of certain genes (MYH16) may be related to the reduction of the diameter of type II fibers [25]. In addition to these differences and given the more complex functions that are performed by these muscles in non-human primates, such as language, it is presumed that the structure of the jaw-elevator muscles is more complex in humans than in primates. This complexity could indicate the existence of functional compartmentalization.

The various levels of activation of the MUs located in the different SM recording sites (A, MA, MP and P) could be related to a functional specialization of MUs depending on their location. This functional specialization may be related to the heterogeneous distribution of the type of muscle fibers within the SM. In masticatory muscles of animals, it has been observed that in the most anterior and deepest regions, they contain more type I muscle fibers, while the more superficial and posterior regions contain more type IIa fibers [26]. However, the topic is controversial in humans. While some researchers have reported that the SM has a larger number of type IIa fibers [27], others argue that there would be a predominance of hybrid fibers [28]. Furthermore, there are reports of a higher percentage of type I fibers in the anterior portion of the SM [29]. Our results are consistent with those obtained previously by other authors [3], given that during the bite at submaximal force, the MUs of the anterior part were more recruited at low levels of the masticatory force than the MUs located in the posterior portion. This would indicate that MUs of the SM would have different activation thresholds depending on their location in the muscle. Previously, the differences in the activation thresholds of MUs have been explained by the size principle [30], which describes the recruitment of MUs during the development of progressive muscle force. At low force levels, the small MUs (motoneurons of small soma size with fewer muscular fibers) are first recruited, while larger MUs (motoneurons of larger soma size with more muscular fibers) are recruited as the demand of force is increased. In most skeletal muscles, small MUs have slow contraction fibers or type I fibers, while large MUs are formed by fast contraction fibers or type II fibers [20]. Our results indicate that the MUs of the anterior portion of the SM have a lower activation threshold than those of the posterior portion, which according to the size principle, suggests that the MUs located in the anterior portion of the SM would be small MUs. In this direction, histological studies have shown that type I muscle fibers are predominant in the anterior portion of the SM [29]. Interestingly, these muscle fibers are larger in diameter than type II fibers [27, 29]; however, it has been established that this larger size of the slow fibers does not imply a larger size of these motor units [31]. To that end, our results would indicate that the recruitment of MUs in the SM would not be in accordance with the size principle. Additionally, there is evidence that suggests that in some special situations, the recruitment of MUs in the SM would not correspond to the size principle [32]; therefore, there is a need for future research on the recruitment strategies of the MUs in the SM under different conditions. Our results indicate that the MUs of the SM are progressively recruited from the anterior towards the posterior portion. This assertion is supported by the COMx behavior during the different levels of BF (Fig. 1c). At low levels of BF, the COMx was located towards the anterior portion of the SM, and it moved towards the posterior portion as BF increased. This can be interpreted as an increase in the recruitment of the MUs located in the posterior portion of the SM when the demand of BF increases. This conclusion implies that at low levels of BF, there is greater variation in recruiting MUs, and as the demand of BF increases, the MUs’ activity becomes more homogeneous. This statement is supported by the entropy index behavior (Fig. 1b), which is consistent with that previously reported by other authors [33].

One of the major limitations of this research was the sampling of a cohort of extremely healthy individuals, which is not a common factor in the general population, from a dental perspective. Thus, we used a small sample consisting of young, healthy subjects with an ideal craniofacial system growth and development. While perhaps our results cannot be extrapolated to the general population, the selection of this sample allowed for the control of confounding factors associated with adaptive and/or pathological states of the masticatory system.

Regional differences in the level of activation were mainly observed at low levels of BF and between the MUs located in A and P, not between those located in MA and MP; this could be attributed to the fact that MUs located in these regions would be MUs with intermediate functional characteristics compared to those located in A and P.

The results of this study show that motor units of the SM have different levels of activation in relation to their location and developed BF level. The findings suggest that the superficial masseter is organized into three functional compartments: an anterior, a middle and a posterior compartment; however, this compartmentalization is only seen at low levels of BF (20–60% MVBF).
